# Human metapneumovirus driven IFN-β production antagonizes macrophage transcriptional induction of IL1-β in response to bacterial pathogens

**DOI:** 10.3389/fimmu.2023.1173605

**Published:** 2023-06-26

**Authors:** Simon Loevenich, Nicola P. Montaldo, Arthur Wickenhagen, Tatyana Sherstova, Barbara van Loon, Victor Boyartchuk, Marit W. Anthonsen

**Affiliations:** ^1^ Department of Clinical and Molecular Medicine, Norwegian University of Science and Technology (NTNU), Trondheim, Norway; ^2^ Department of Biotechnology and Nanomedicine, SINTEF Industry, Trondheim, Norway; ^3^ Department of Viroscience, Erasmus Medical Center, Rotterdam, Netherlands; ^4^ Clinic of Surgery, St Olav Hospital HF, Trondheim, Norway

**Keywords:** human metapneumovirus, co-infection, airway bacteria, proinflammatory cytokines, inflammasome, IFN-β, IL-1β

## Abstract

Human metapneumovirus (HMPV) is a pneumovirus that may cause severe respiratory disease in humans. HMPV infection has been found to increase susceptibility to bacterial superinfections leading to increased morbidity and mortality. The molecular mechanisms underlying HMPV-mediated increase in bacterial susceptibility are poorly understood and largely understudied. Type I interferons (IFNs), while critical for antiviral defenses, may often have detrimental effects by skewing the host immune response and cytokine output of immune cells. It is currently unknown if HMPV skews the inflammatory response in human macrophages triggered by bacterial stimuli. Here we report that HMPV pre-infection impacts production of specific cytokines. HMPV strongly suppresses IL-1β transcription in response to LPS or heat-killed *Pseudomonas aeruginosa* and *Streptococcus pneumonia*, while enhancing mRNA levels of IL-6, TNF-α and IFN-β. We demonstrate that in human macrophages the HMPV-mediated suppression of IL-1β transcription requires TANK-binding kinase 1 (TBK1) and signaling via the IFN-β-IFNAR axis. Interestingly, our results show that HMPV pre-infection did not impair the LPS-stimulated activation of NF-κB and HIF-1α, transcription factors that stimulate IL-1β mRNA synthesis in human cells. Furthermore, we determined that sequential HMPV-LPS treatment resulted in accumulation of the repressive epigenetic mark H3K27me3 at the *IL1B* promoter. Thus, for the first time we present data revealing the molecular mechanisms by which HMPV shapes the cytokine output of human macrophages exposed to bacterial pathogens/LPS, which appears to be dependent on epigenetic reprogramming at the *IL1B* promoter leading to reduced synthesis of IL-1β. These results may improve current understanding of the role of type I IFNs in respiratory disease mediated not only by HMPV, but also by other respiratory viruses that are associated with superinfections.

## Introduction

Human metapneumovirus (HMPV) is an enveloped negative-sense, single-stranded RNA virus that belongs to the *Pneumoviridae* family (1). In the elderly, young children and patients with underlying respiratory disease or in immunosuppressed individuals, HMPV can cause severe disease and high mortality (2). There are currently no targeted treatments or vaccines available for HMPV.

Severe viral respiratory disease is often associated with secondary bacterial infections that can lead to pneumonia with high morbidity and mortality (3). For influenza virus, secondary bacterial infections have been suggested to be responsible for most deaths during pandemic outbreaks (4). Secondary bacterial infections are less commonly documented with noninfluenza respiratory viruses, including HMPV. However, recent epidemiological studies have shown that frequencies and types of bacterial coinfections with noninfluenza respiratory viruses are similar to those for influenza virus (5). Clinical observations indicate that infections with HMPV and the highly related respiratory syncytial virus (RSV) are associated with a higher risk for bacterial coinfections and bacterial pneumonia (6–9).

Though it is clear that respiratory viral infection can modulate subsequent bacterial infections, relatively little is known about the molecular mechanisms that mediate altered responses to bacteria. Nevertheless, it has been found that attenuated barrier function of the respiratory epithelium, decreased recruitment of neutrophils and altered ability of dendritic cells to respond to pathogen-associated molecular patterns are linked to increased bacterial susceptibility after viral infection (10, 11). Moreover and importantly, an altered expression of proinflammatory cytokines and distorted macrophage cytokine output have been reported as key contributing factors and mechanisms for these severe dual infections for influenza virus with bacteria (12, 13). For noninfluenza respiratory viruses such as HMPV, the mechanisms that lead to increased susceptibility remain largely unknown and understudied, with few or no reports on human primary cells or models for HMPV.

Type I interferons (IFNs) are critical for antiviral defense mechanisms by triggering expression of IFN-stimulated genes (ISGs) that limit viral replication. However, type I IFNs may also lead to immunosuppression, a process that is instrumental during the resolution phase of infection and contribute to enhanced host susceptibility to secondary bacterial infections (11, 14). Interestingly, it has been reported that after influenza virus infection IFNs may suppress activation of the inflammasome leading to reduced levels of mature interleukin (IL)-1β protein and impaired antibacterial activity of neutrophils (15, 16).

IL-1β is a potent, multifunctional proinflammatory cytokine that is primarily produced by monocytes and macrophages and controls a variety of immunological functions such as proliferation, activation and recruitment of inflammatory cells (17). It is established that IL-1β plays a critical role in antimicrobial host defense and protection against such bacteria as *S. pneumoniae* or *P. aeruginosa* (18, 19). Studies in mice and humans have shown that in the case of the influenza virus, reduced IL-1β levels are associated with susceptibility to secondary bacterial infection (12, 20). Decrease in IL-1β levels results in impaired type 17 immunity and neutrophil function, thereby lowering antibacterial defense mechanisms (12, 20). Because IL-1β has a crucial regulatory role in the overall inflammatory state of the host, the activity of IL-1β is tightly controlled at the levels of transcription, translation, maturation, and proteasomal degradation (21–23) processes that can all be regulated to tailor host response to different pathogens.

In this study, we set out to establish how HMPV infection of human primary macrophages affects the subsequent innate immune response to LPS or heat-killed airway bacteria. Our results demonstrate that HMPV uses a TBK1- IFN-β-IFNAR-driven mechanism to differentially control transcription of specific cytokines and reduce IL-1β transcription in response to LPS or heat-killed *Pseudomonas aeruginosa* and *Streptococcus pneumonia*.

## Materials and methods

### Virus propagation

Sendai virus (Cantell strain) was purchased from Charles River Laboratories. The clinical HMPV isolate NL/17/00 (A2) was kindly provided by ViroNovative and B. van den Hoogen (Erasmus MC, Rotterdam). LLC-MK2 cells were inoculated with HMPV at a multiplicity of infection (MOI) of 0.01 in OptiMEM containing 2% FBS, 20 µg/mL gentamicin and 0.7 nM glutamine. After 7-8 days, the virus was harvested by freeze-thawing at -80 °CC, followed by purification on a 20% sucrose cushion and resuspension in OptiMEM (2% FBS). The virus titer was determined using a cell-based immunoassay. To that end, purified virus was serially diluted (log10) on monolayers of LLC-MK2 cells in 96-well flat-bottom plates. After four days, cells were washed, stained with LIGHT DIAGNOSTICS™ HMPV direct fluorescence assay (Merck Millipore) and foci forming units determined by manual counting. Hep-2 cells were inoculated with RSV at a MOI of 0.01 in DMEM (2% FBS, 100 U/mL Penicillin, 100 U/mL Streptomycin, 0.7 nM glutamine). After 2 days, culture medium was changed. The virus was harvested on the third day by freeze-thawing and was purified by ultra-centrifugation on a 60% sucrose cushion, followed by ultra-centrifugation on a discontinuous gradient of 60% and 30% sucrose. The purified virus was diluted 1:1 in OptiMEM containing 2% FBS. Viral titers were determined as for HMPV. For inactivation, virus was irradiated with UV light for 1 h at 4°C.

### Cell culture

Monocytes were isolated from fresh buffy coats of healthy donors (blood bank of St. Olavs Hospital, Trondheim). In short, mononuclear cells were isolated using gradient centrifugation with Lymphoprep™ (Axis-Shield). Isolated cells were seeded in RPMI 1640 medium supplemented with 10% human serum, 0.34 mM L-glutamine, 10 µg/mL gentamicin. After 90 min non-adherent cells were washed away. Monocytes were differentiated to macrophages for 14 days in RPMI 1640 supplemented with 10% human serum, 0.34 mM L-glutamine, 10 µg/mL gentamicin and 10 ng/mL macrophage colony-stimulating factor. A549 cells were cultivated in supplemented RPMI 1640 (10% FBS, 0.7 nM L-glutamine, 20 µg/mL gentamicin). THP-1 ASC-GFP cells were differentiated with PMA as previously described (24).

### *In vitro* HMPV infection

Cells were infected with HMPV A2 or RSV A2 at multiplicity of infection (MOI) 1 or with SeV (100 hau) in OptiMEM containing 2% FBS, 20 µg/mL gentamicin and 0.7 nM glutamine. Cells were incubated with virus for the indicated times.

### Stimulation of cells with bacterial ligands

DH5α E. coli were heat-killed at 70°C for 40 minutes. Heat-killed clinical isolates of *P. aeruginosa* and *S. pneumoniae* were kindly provided by Svein Arne Nordbø and Andreas Christensen (NTNU/St. Olavs Hospital, Trondheim). Cells were stimulated with either 500 ng/mL LPS from E. coli O111:B4 (Sigma), 10^5 cell/mL heat-killed DH5α E. coli, 1:10 diluted isolate of *P. aeruginosa* or 1:10 diluted isolate of *S. pneumoniae*. For analysis of gene expression and transcription factor levels, cells were stimulated for 2 h. For analysis of IL-1β maturation, cells were stimulated 13 h followed by 10 µg/mL nigericin for 40 min (Sigma).

### Recombinant interferons, neutralizing antibodies and inhibitors

Anti-human IFN- β, recIFN-α and -β were purchased from Peprotech and nIFNAR MMHAR-2 from PBL. Cells were preincubated with the TBK1 inhibitor BX795 (5 µM) or the IFNAR-blocking antibody nIFNAR (10 µg/mL) 30 min before infection with virus. For neutralization of secreted IFN-β, 20 µg/mL nIFN-β antibody were added simultaneously with virus. RecIFN-β (1000 U/mL) was added 6 h after infection with virus for a total of 12 h.

### Supernatant transfer

Human macrophages were pretreated with or without BX795, then incubated with HMPV (MOI=1) for 24 h. Supernatants were retrieved after HMPV incubation. Macrophages were either directly harvested or treated with LPS. The collected supernatants were filtered (cut-off 0,1 µm) and transferred to untreated macrophages. These macrophages were incubated in the conditioned medium (CM) for 12 h, followed by either direct harvest or treatment with LPS.

### Chromatin immunoprecipitation

Cultured macrophages were crosslinked in 1% formaldehyde. Cross-linking was quenched by adding glycine to a 0.110 mM concentration. Cells were washed and harvested in ice-cold PBS. Cells were resuspended with cell lysis buffer (100mM Tris-HCl pH 8, 10 mM DTT) and incubated for 15 mins on ice and 15 min at 30°C shacking. The lysed cells were then centrifuged to pellet nuclei. After washing twice (1st wash: 10 mM EDTA, pH 8, 10 mM EGTA, 10 mM HEPES pH 8, 0.25% Triton X; 2nd wash: 10 mM EDTA, pH 8, 0.5 mM EGTA, 10 mM HEPES pH 8, 200 mM NaCl), the nuclear pellets were lysed in 300 μl of Nuclear lysis buffer (50 mM Tris-HCl pH 8, 10 mM EDTA and 1% SDS) and fragmented by sonication using a Bioruptor ultrasonic cell disruptor (Diagenode) for 30 cycles of 30 seconds. Sheared chromatin to an average fragment size of 200-250 bp was evaluated via agarose gel electrophoresis. 20 μg of chromatin was first precleared for 2 hours at 4°C and then immunoprecipitated in ChIP buffer (16.7 mM Tris-HCl pH 8, 167 mM NaCl, 1.2 mM EDTA, 0.01% SDS and 1.1% Triton X-100) with 2 μg of antibody overnight at 4°C. The complexes DNA-protein-antibodies were isolated using A/G dynabeads (ThermoFisher Scientific). Washes were performed using low salt wash buffer (16.7 mM Tris-HCl pH 8, 167 mM NaCl 0.1% SDS, 1% Triton X), high salt wash buffer (16.7 mM Tris-HCl pH 8, 500 mM NaCl 0.1% SDS, 1% Triton X) and LiCl wash buffer (250 mM LiCl, 0.5% NP40, 0.5% Na-deoxycholate, 1mM EDTA, 10mM Tris-HCl pH 8). Proteinase K treatment was performed for 1 hr at 50°C with 10 mM EDTA, 40 mM Tris-HCl pH 6.5 and 20 μg proteinase K. The chromatin was purified with phenol-chloroform and ethanol precipitated. Input DNAs were used as templates for qRT-PCR. Final concentrations are shown as relative input. Relative enrichment was calculated as follows: Target sequence enrichment for each single experiment was calculated as percentage of input and normalized against one consistent sample (LPS GAPDH -400bp) prior to calculating the mean of all experiments. Antibodies for H3K27me3 (C15410195) and rabbit IgG (C15410206) were purchased from Diagenode. The following primers were used: IL1B -250 fwd CAAATGTATCACCATGCAAATATGC, IL1B -250 rev CGTGGGAAAATCCAGTATTTTAATG, GAPDH -400 Fwd GTGCGTGCCCAGTTGAACC, GAPDH -400 rev CTTGAGGCCTGAGCTACGTG, TNF -98 fwd ACTACCGCTTCCTCCAGATGAG and TNF -98 rev GGGAAAGAATCATTCAACCAGCGG.

### qRT-PCR analysis

RNA isolation, cDNA synthesis and qRT-PCR analysis were performed as previously described (25). Relative gene expression was calculated according to Livak et al. (26). For analysis of HMPV and RSV vRNA expression, the following primers were used (sequences 5’-3’): HMPV N-gene (fwd) CATATAAGCATGCTATATTAAAAGAGTCTC, HMPV N-gene (rev) CCTATTTCTGCAGCATATTTGTAATCAG. Fold-change in HMPV vRNA expression was calculated relative to the indicated virus sample. All other primer sequences have been published previously (25).

### Immunoblotting

Preparation of whole-cell lysates was performed as described previously (27). SDS-PAGE and immunoblotting of whole cell lysates were performed as previously described (27). Band intensities were normalized against medium and loading control (β-actin/GAPDH) using Image Studio (Licor). In case of phophoproteins additional normalization against total target protein levels was performed. Nuclear fractionation of samples was performed based on a previously published protocol with modifications (28). Cells were detached by trypsination followed by scraping. Cells were washed in 30 volumes of PBS and centrifuged (5 min, 450 x g). The cell pellet was resupended in one packed cell volume (PCV) of ice cold Buffer A + DTT with minimal pipetting and allowed to swell on ice for 15 min. The cells were lysed by slowly drawing the cell suspension into a 1 ml syringe with a 25-g 5/8 gauge needle and then rapidly expelling in a single stroke. This was repeated 5 times. The homogenate was centrifuged for 5 min at 12,000 x g at room temperature, yielding a crude nuclear pellet and a post nuclear supernatant. The post nuclear supernantant was processed to make a cytoplasmic extract by adding 0.11 PCV of Buffer B and spinning 5 min at 12,000 x g and 4° C. The crude nuclear pellet was resuspended in 0.67 PCV of ice cold Buffer C containing 420 mM NaCl, then incubate on ice for 30 min. Nuclear debris was removed by centrifugation (5 min, 12.000 x g, 4° C). The supernatant containing the nuclear extract was snap-frozen in liquid nitrogen and stored at -80°C. SDS-PAGE and immunoblotting of nuclear fractions were performed as for whole cell lysates. Band intensities were normalized against medium and loading control (Histone 3) using Image Studio (Licor). The following primary antibodies were used: β-actin (Sigma Aldrich), GAPDH (Cell Signaling Technology), HIF-1α (Abcam), Histone 3 (Abcam), IκBα (Cell Signaling Technology), IκBβ (Cell Signaling Technology), IL-1β (Cell Signaling Technology), IRF3 (Cell Signaling Technology), p-IRF3(S396) (Cell Signaling Technology), p65, p-p65(Ser536) (Cell Signaling Technology), p-TBK1(Ser172) (Cell Signaling Technology), TBK1 (Cell Signaling Technology).

### ELISA

Levels of secreted IL-1β in culture supernatants were determined using the IL-1β OptEIA™ Set (BD Biosciences) following the manufacturer’s instructions.

### Confocal microscopy

THP-1 ASC-GFP cells were incubated with HMPV (MOI=1) for 18 h followed by LPS (2h) and nigericin. Cells were fixed with 4% PFA (10 min), nuclei stained with DAPI and the proportion of speck containing cells determined by confocal microscopy.

### Statistical analysis

Statistical differences for multiple comparisons were calculated by one- or two-way ANOVA followed by *post hoc* Tukey’s multiple comparisons test. Unpaired Student’s t-test was used for statistical analysis of single comparisons. Differences were considered significant when p ≤ 0.05 (∗), very significant when p ≤ 0.01 (∗∗), and extremely significant when p ≤ 0.001 (∗∗∗).

## Results

### HMPV suppresses IL-1β secretion and transcription in response to LPS and airway bacteria

**Figure 1:**
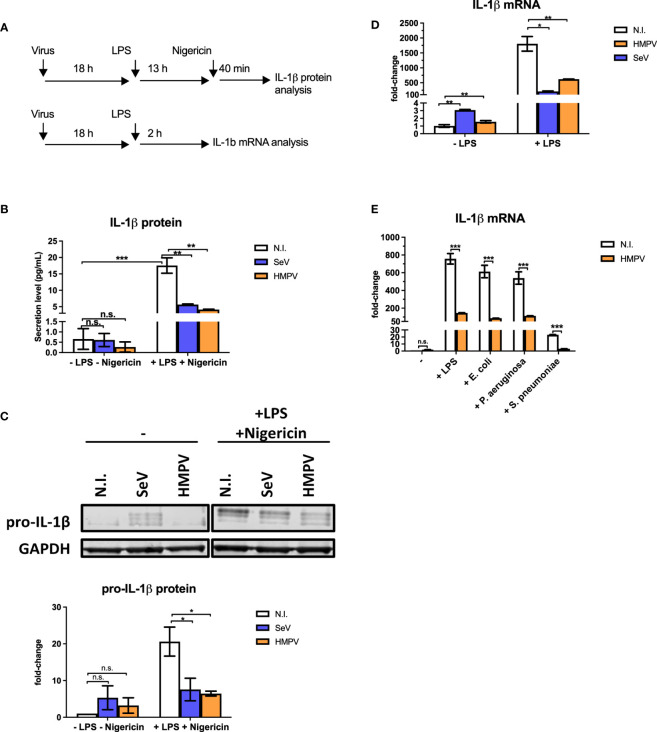
Preinfection of human macrophages with HMPV impairs IL-1β expression by LPS and bacterial ligands. **(A)** Schematic presentation of the experimental setup. Human macrophages were infected with virus before stimulation with LPS (for mRNA analysis) or LPS and nigericin (for protein analysis). **(B, C)** Human macrophages were incubated with HMPV, SeV or medium (N.I. = not infected) and then stimulated with LPS for 13 h and nigericin for 40 min. Culture supernatants were analyzed for secreted IL-1β by ELISA **(B)** and total cell lysates were analyzed for pro-IL-1β protein levels by immunoblotting **(C)**. In **(C)** pro-IL-1β protein levels were normalized against GAPDH. Error bars represent SD of two independent biological replicates. **(D)** Human macrophages were incubated with HMPV, SeV or medium (N.I. = not infected) and then stimulated with LPS for 2 (h) IL-1β mRNA expression was analyzed by qRT-PCR. Error bars represent SD of three technical replicates. Data are representative for at least eight (HMPV) or two (SeV) biological replicates. **(E)** Human macrophages were incubated with HMPV and then stimulated with either LPS, heat-killed E. coli, heat-killed P. *aeruginosa* or heat-killed S. *pneumoniae* for 2 (h) IL-1β mRNA expression was analyzed by qRT-PCR. Error bars represent SD of three technical replicates. Data are representative for two independent biological replicates. Statistical analysis: two-way ANOVA with *post hoc* Tukey’s multiple comparisons test: ∗p < 0.05; ∗∗p < 0.01; ∗∗∗p < 0.001; n.s., not significant.

To determine if HMPV preinfection affects production of the antibacterial IL-1β cytokine we infected human monocyte-derived macrophages (MDMs) with HMPV or Sendai virus (SeV) for 18 hours prior to stimulation with LPS. SeV was chosen for comparison as it is a strong inducer of RIG-I-signaling and IFN-β production (29). Initially, we determined the effect of viral preinfection on LPS-stimulated IL-1β secretion into the extracellular medium (the experimental outline is shown in **Figure 1A**). Incubation with either HMPV or SeV alone failed to significantly increase levels of mature IL-1β in the supernatant of human macrophage cell culture (**Figure 1B**, left bars), despite the fact that virus indeed replicates at this timepoint of infection (**Figure S1**). As expected, LPS/nigericin treatment strongly increased the production of mature IL-1β in the medium as shown in previous reports [**Figure 1B**, (30)]. However, in macrophages that had been pre-infected with HMPV or SeV extracellular IL-1β was strongly reduced upon LPS/nigericin-treatment (**Figure 1B**), suggesting that HMPV and SeV impair LPS-triggered release of mature IL-1β.

Bioactive IL-1β synthesis is regulated in multiple steps; transcription, translation, proteasome and caspase processing and secretion of mature IL-1β (23, 30).

To address if HMPV preinfection affected the step of pro-IL-1β processing by the inflammasome, we studied formation of ASC (apoptosis-associated speck-like protein containing a CARD) specks as a marker of inflammasome activation. ASC is a scaffold component of different inflammasomes and the formation of ASC specs promotes caspase-1-mediated processing of pro-IL-1β (30). For this purpose we made use of a THP-1 cell line stably expressing GFP-tagged ASC and monitored speck formation by confocal microscopy as previously described (24). We found that LPS and nigericin induced ASC specks in about 42% of THP-1 cells (**Figure S2A**). HMPV infection alone did not induce speck formation and preinfection with HMPV did not change the proportion of speck-containing cells following stimulation with LPS/nigericin (**Figure S2**). Hence, LPS/nigericin-triggered ASC speck formation, reflecting upstream inflammasome activation, was not impaired by HMPV preinfection of THP-1 cells.

IL-1β is produced intracellularly as an inactive precursor, termed pro-IL-1β (generally referred to as the priming step). To examine if HMPV or SeV affected the intracellular pool of pro-IL-1β in a similar manner as secreted IL-1β detected by ELISA, whole cell lysates were analyzed by immunoblotting (**Figure 1C**). We found that LPS-triggered expression of pro-IL-1β and that levels of LPS-triggered IL-1β was strongly decreased in human MDMs pre-infected with a virus (**Figure 1C**). Hence, preinfection with HMPV and SeV affects IL-1β production at a step prior to pro-IL-1β synthesis and secretion of mature IL-1β.

To determine if reduced intracellular pools of IL-1β in HMPV-LPS-treated cells could be due to reduced IL-1β transcription, we examined IL-1β mRNA levels by RT-qPCR analysis. Indeed, the LPS-stimulated IL-1β mRNA levels were markedly reduced in human macrophages that had been infected with HMPV or SeV prior to treatment (**Figure 1D**). Consistent with IL-1β protein measurements, virus alone induced only small amounts of IL-1β mRNA (**Figure 1D**, left bars). Thus, collectively, our results show that HMPV impairs LPS/bacteria-triggered IL-1β production at the transcriptional level.

We next confirmed that the effect of HMPV preinfection on IL-1β mRNA expression was similar for airway bacteria to that for purified LPS. Human MDMs were treated with heat-killed *E. coli, P. aeruginosa* and *S. pneumoniae*, human pathogens that are physiologically relevant for secondary bacterial infections. Similar to LPS, heat-killed *E. coli, P. aeruginosa* and *S. pneumoniae* induced IL-1β mRNA and this induction was strongly reduced in MDMs that had been pre-infected with HMPV, (**Figure 1E**). Hence, HMPV infection of MDMs has similar repressive effect on IL-1β transcription in response to both LPS and heat-killed airway bacteria.

### HMPV specifically reduces IL-1β mRNA while increasing TNF-α, IL-6 and IFN-β transcript induction by bacterial triggers

**Figure 2:**
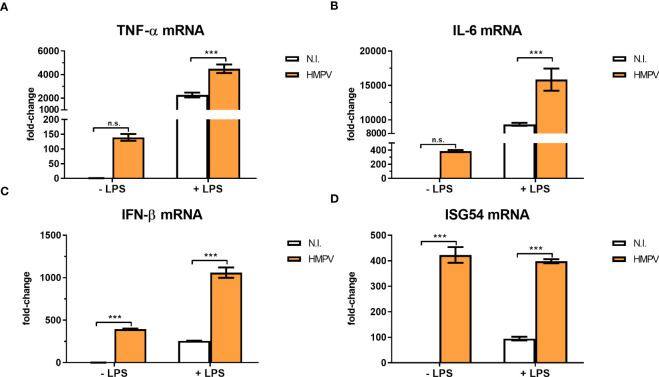
HMPV infection enhances LPS-induced expression of TNF-α, IL-6, IFN-β and ISG54. **(A–D)** Human macrophages were incubated with HMPV or medium (N.I. = not infected) before stimulation with LPS for 2 h and analysis of mRNA expression of TNF-α, IL-6, IFN-β and ISG54 by qRT-PCR. Error bars represent SD of three technical replicates. Data are representative for at least two biological replicates. Statistical analysis: two-way ANOVA with *post hoc* Tukey’s multiple comparisons test ∗∗∗p < 0.001; n.s., not significant.

The coordinated expression of cytokines is essential for the host response to pathogen infections and altered expression of proinflammatory cytokines has been reported as a key contributing factor to secondary infections (13). To characterize the impact of HMPV on transcription of a subset of relevant cytokines and antiviral genes, we measured mRNA levels of TNF-α, IL-6, IFN-β and ISG54 (IFIT2) in human macrophages treated with LPS subsequent to HMPV infection. LPS treatment alone induced marked amounts of TNF-α, IL-6, ISG54 and IFN-β mRNA. In contrast, HMPV-stimulated expression levels of TNF-α and IL-6 mRNA were low compared to LPS (**Figures 2A, B**). As expected, HMPV induced IFN-β and ISG54 mRNA expression to much higher extent than LPS (**Figures 2C, D**). Interestingly, in MDMs that had been preinfected with HMPV prior to LPS stimulation the induction of TNF-α, IL-6 and IFN-β mRNA expression was significantly enhanced compared to cells treated only with LPS (**Figures 2A–C**), hence showing opposite response to that of IL-1β mRNA.

**Figure 3:**
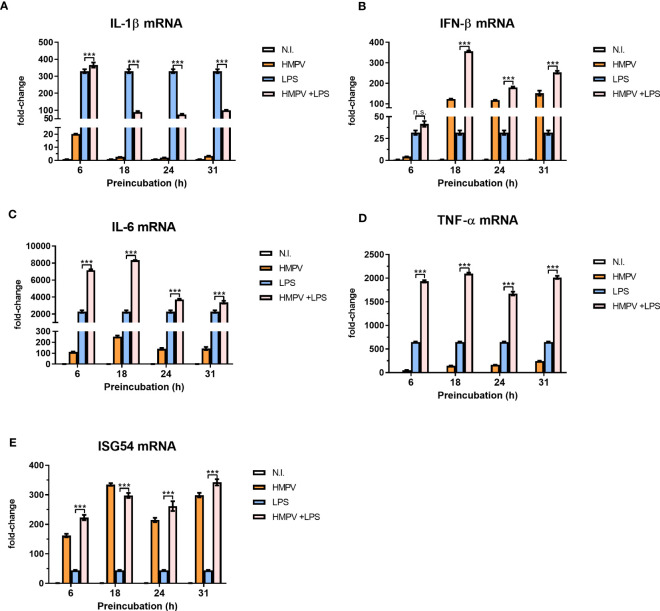
Progression of HMPV infection determines the viral effect on proinflammatory cytokines induced by LPS. **(A–E)** Human macrophages were incubated with HMPV or medium for the indicated time-points before stimulation with LPS for 2 h and analysis of mRNA expression of IL-1b, IFN-b, IL-6, TNF-a and ISG54 by qRT-PCR. Error bars represent SD of three technical replicates. Data are representative for two biological replicates. Statistical analysis: two-way ANOVA with *post hoc* Tukey’s multiple comparisons test: ∗ ∗ ∗p < 0.001; n.s., not significant.

To determine how progression of HMPV replication altered the outcome of LPS-induced proinflammatory cytokines, we preinfected macrophages with HMPV for 6, 18, 24, or 31 hours prior to stimulations with LPS. We found that the inhibitory effect of HMPV on LPS-triggered IL-1β mRNA expression became detectable between 6 and 18 hours post viral infection (**Figure 3A**). Indeed, by monitoring HMPV viral RNA (at different timepoints) we observed that HMPV vRNA increased between 6 and 18 hours (**Figure S1**). In contrast to IL-1β mRNA (for which HMPV inhibited the induction by LPS), we observed an additive effect of HMPV preinfection on LPS-stimulated IFN-β mRNA levels, an effect that became apparent at 6 to 18 hours post viral infection (**Figure 3B**). Similar to IFN-β, mRNA expression of IL-6 and TNF-α was synergistically induced by LPS and HMPV (**Figures 3C, D**). In contrast, ISG54 mRNA expression was similar in HMPV-infected and HMPV+LPS-treated cells (**Figure 3E**). Overall, these results show that HMPV preinfection has distinct dynamic effects on specific cytokines. It synergistically enhances LPS-stimulated IFN-β, IL-6 and TNF-α transcription, while blocking IL-1β mRNA induction.

### The effect of HMPV on LPS-triggered responses is dependent on viral replication and the innate immune kinase TBK1

**Figure 4:**
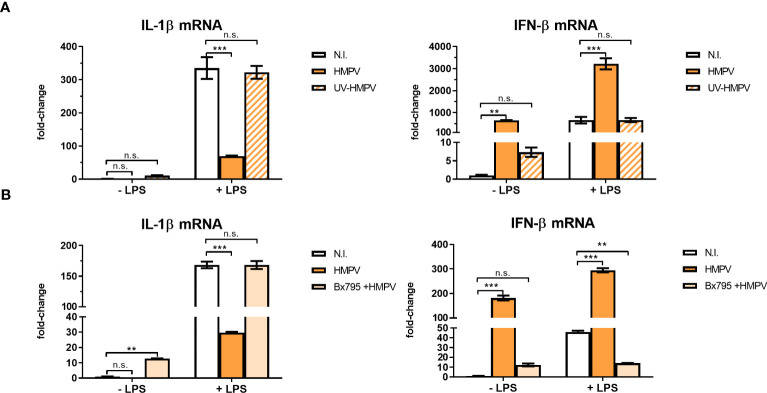
Viral replication and TBK1 activity are required for the capacity of HMPV to repress LPS-induced transcription of IL-1β. **(A)** Human macrophages were incubated with HMPV, UV-inactivated HMPV or medium (N.I. = not infected) before stimulation with LPS for 2 (h) Expression of IL-1β and IFN-β mRNA was analyzed by qRT-PCR. Error bars represent SD of three technical replicates. Data are representative for at least two biological replicates. **(B)** Human macrophages were pretreated with or without the semi-selective TBK1/IKKε inhibitor BX795, then incubated with HMPV before stimulation with LPS (2 h). Expression of IL-1β and IFN-β mRNA was analyzed by qRT-PCR. Error bars represent SD of three technical replicates. Data are representative for at least two biological replicates. Statistical analysis: two-way ANOVA with *post hoc* Tukey’s multiple comparisons test: ∗∗p < 0.01; ∗∗∗p < 0.001; n.s., not significant.

To evaluate if HMPV replication was required for limiting LPS-stimulated IL-1β expression, we explored the effect of UV-treated, non-replicating HMPV on IL-1β and IFN-β mRNA expression by LPS. We confirmed that UV-treated HMPV was unable to replicate (**Figure S3**). Furthermore, in contrast to live virus, UV-inactivated HMPV failed to reduce LPS-triggered IL-1β expression and did not stimulate IFN-β (**Figure 4A**, left panel).

Next, we examined the effect of inhibiting TBK1, the kinase responsible for phosphorylation of the transcription factor IRF3 on inhibition of IL-1β transcription in HMPV-LPS treated MDMs. The TBK1-IRF3 axis is critical for induction of IFN-β in several cell types including macrophages (31, 32). We found that the semi-selective TBK1/IKKε inhibitor BX795 reversed the effect of HMPV on LPS-induced IL-1β mRNA expression in macrophages (**Figure 4B**, left panel). Also, HMPV-stimulated IFN-β mRNA induction was blocked by BX795 (**Figure 4B**, right panel). These data suggest that HMPV replication and TBK1-mediated signaling is required for the ability of HMPV to reduce IL-1β transcription by LPS/bacterial triggers.

### IFN-β mediates the suppressive effects of HMPV infection on IL-1β transcription

**Figure 5:**
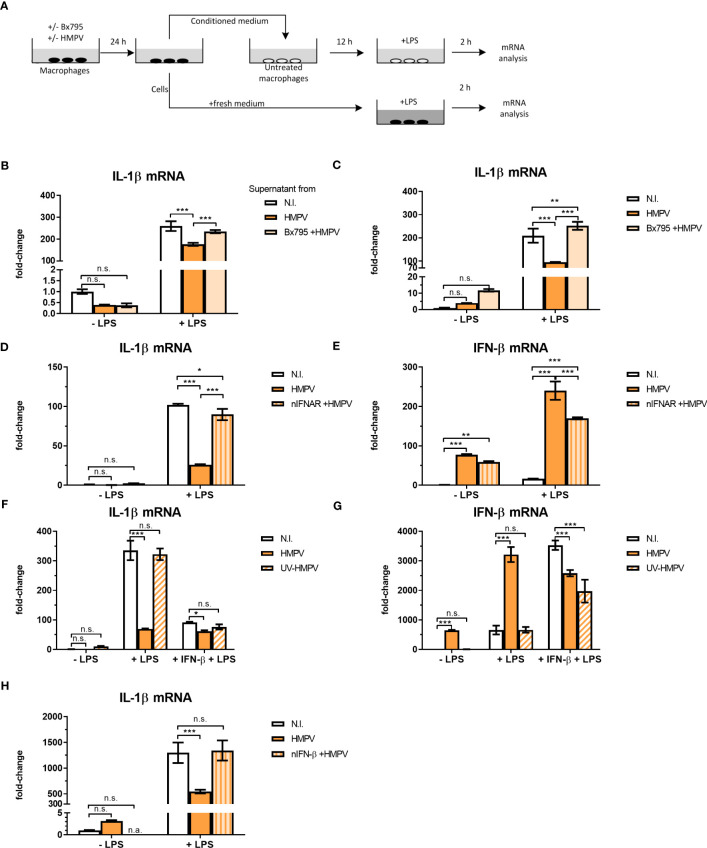
HMPV-induced repression of LPS-triggered IL-1β transcription is mediated by IFN-β. **(A)** Workflow of supernatant transfer experiments. Human macrophages were pretreated with or without BX795 and incubated with HMPV for 24 h. Supernatants were retrieved after HMPV infection. Macrophages were either directly harvested or treated with LPS for 2 h. The collected supernatants were filtered and transferred to untreated macrophages. These macrophages were incubated in the conditioned medium (CM) for 12 h, followed by either direct harvest or treatment with LPS for 2 h. **(B, C)** “CM-donor” macrophages **(C)** and CM-treated macrophages **(B)** were analyzed for IL-1β mRNA expression by qRT-PCR. Error bars represent SD of three technical replicates. **(D, E)** Human macrophages were pretreated with or without nIFNAR, then incubated with HMPV before stimulation with LPS for 2 h prior to determination of IL-1β **(D)** or IFN- β **(E)** mRNA expression by qRT-PCR analysis. Error bars represent SD of three technical replicates. Data are representative for at least two biological replicates **(F, G)** Human macrophages were incubated with HMPV or UV-inactivated HMPV. Recombinant IFN-β was added to the indicated samples 12 h before stimulation with LPS for 2 (h) Expression of IL-1β **(F)** and IFN-β mRNA **(G)** was analyzed by qRT-PCR. Error bars represent SD of three technical replicates. Data are representative for at least two biological replicates. **(H)** Human macrophages were incubated with HMPV in presence or absence of nIFN-β before stimulation with LPS for 2 (h) Expression of IL-1β mRNA was analyzed by qRT-PCR. Data are representative for two biological replicates. Statistical analysis: two-way ANOVA with *post hoc* Tukey’s multiple comparisons test: ∗p < 0.05; ∗∗p < 0.01; ∗∗∗p < 0.001; n.s., not significant. “n.a.” not analyzed.

To explore if the inhibitory effect of HMPV on LPS-induced IL-1β was mediated by secreted factors, MDMs (“donor MDMs”) were treated with the TBK1/IKKε inhibitor BX795, infected withHMPV and conditioned medium (CM) was harvested. CM was added to human MDMs (“acceptor MDMs”) and incubated for 12 hours prior to LPS-addition and qPCR analysis of IL-1β mRNA expression (experimental setup is schematically depicted in **Figure 5A**). We also analyzed the expression of IL-1β mRNA in MDMs from which the CM was harvested (“donor-MDMs”). We found that CM from HMPV-infected cells significantly reduced LPS-triggered IL-1β mRNA expression (**Figure 5B**), though not as drastically as “direct HMPV infection” did (as shown by analysis of “donor MDMs”; **Figure 5C**). In contrast, CM from cells co-treated with the TBK1/IKKε inhibitor prior to HMPV failed to reduce LPS-triggered IL-1β levels (**Figure 5B**). This suggests that a TBK1-dependent secreted factor is required for the inhibitory effect of HMPV on IL-1β expression.

To test if HMPV-triggered type I IFNs could modulate the inhibitory effect on IL-1β mRNA, human macrophages were preincubated with IFNAR (IFN-α/β receptor) neutralizing antibodies in conjunction with HMPV preinfection and subsequent addition of LPS. Inhibiting signaling of type I IFNs via blocking IFNAR antibodies reversed the effect of HMPV on LPS-induced IL-1β expression (**Figure 5D**). Also, the synergistic effect of HMPV on LPS-stimulated IFN-β mRNA expression was partly reversed by treatment with IFNAR-blocking antibodies (**Figure 5E**). Hence, blocking signaling through IFNAR during HMPV infection restores LPS-stimulated IL-1β expression.

Next, we which type I IFN mediates the observed inhibitory effects by adding recombinant IFN-β in the setting of UV-inactivated HMPV. Recombinant IFN-β significantly reduced LPS-induced IL-1β transcription, with the overall effect being only slightly lower than for live HMPV (**Figure 5F**). Similarly, addition of recombinant IFN-β reduced LPS-triggered IL-1β mRNA expression in cells exposed to UV-HMPV (**Figure 5F**). IFN-β mRNA expression was potently induced by HMPV and LPS alone, but not by UV-HMPV (**Figure 5G**). Recombinant IFN-β exhibited the same synergistic effect on IFN-β induction by LPS as HMPV.

To determine whether the observed effect on IL-1β mRNA expression was mediated exclusively by IFN-β, we used IFN-β neutralizing antibodies. Neutralization of IFN-β blocked the effect of HMPV on LPS-induced IL-1β mRNA expression (**Figure 5H**). Interestingly, we found that at our experimental conditions, pretreatment of cells with recombinant IFN-α did not decrease LPS-induced IL-1β mRNA expression (**Figure S4**). These findings suggest that the effect of HMPV on transcriptional regulation of IL-1β in human macrophages exposed to LPS is largely mediated by secreted IFN-β.

### Preinfection with HMPV does not impair LPS-triggered activation of the NF-κB or HIF-1α pathways

**Figure 6:**
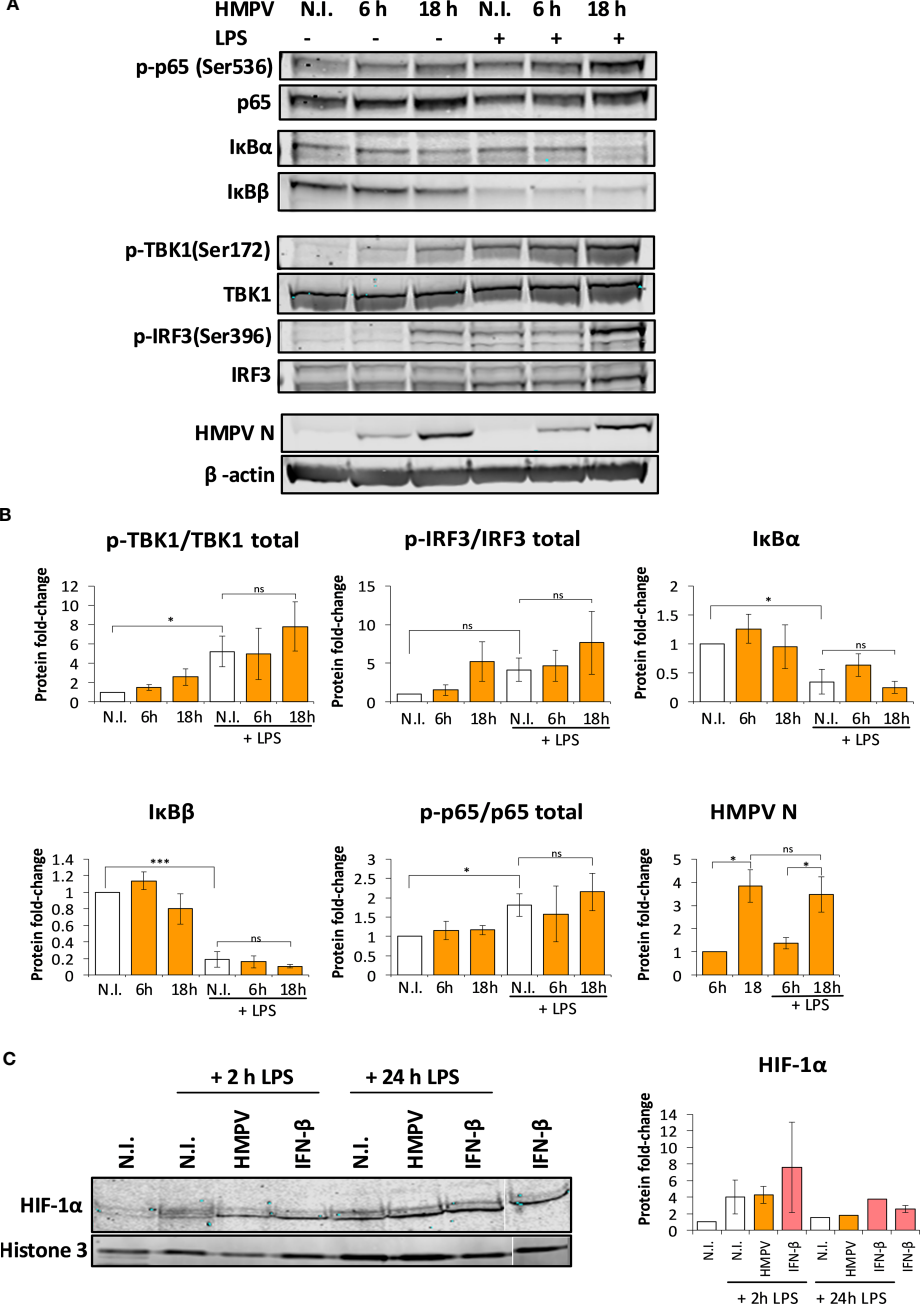
Preinfection with HMPV does not impair LPS-triggered activation of the NF-κB pathway or HIF-1α. **(A, B)** Human macrophages were incubated with HMPV or medium (N.I. = not infected) for the indicated timepoints before stimulation with LPS for 2 h. Whole cell lysates were prepared and activation of signaling molecules was analyzed by immunoblotting. Protein levels were quantified by normalization of band intensities against β-actin. For phosphorylated proteins, protein levels were additionally normalized against total target protein levels. **(A)** p-p65(Ser536), IκBα, IκBβ **(B)** p-TBK1(Ser172), p-IRF3(Ser396) and HMPV Nucleoprotein. The results are expressed as the mean ± SEM of at least 3 independent biological replicates. Statistical analysis: Unpaired Student`s t-test, ∗p < 0.05; ∗∗∗p < 0.001; n.s.: not significant. **(C)** HIF-1α nuclear accumulation upon HMPV infection and LPS treatment. Human macrophages were incubated with HMPV or recombinant IFN-β and then stimulated with LPS for 2 h or 24 h. Cells were lysed, the nuclear fractions enriched and analyzed by immunoblotting. Protein levels in the nuclear fractions were normalized against levels of histone 3. The results are expressed as the mean ± SEM of two independent biological replicates (for 2 hrs LPS and IFN-β). One misloaded sample was cropped from the image in **(C)** and the image was spliced. The full length, uncropped blot is presented in **Figure S5**.

The transcription factor NF-κB stimulates transcription of IL-1β (33). Hence, we were interested to establish if HMPV preinfection impaired the ability of LPS to activate NF-kB. Initially we determined how HMPV preinfection affected p65-Ser536 phosphorylation as phosphorylation at this site is critical for NF-κB transcriptional activity (34). Human MDMs were preinfected with HMPV for either 6 or 18 hours prior to addition of LPS and analysis by immunoblotting. We found that HMPV alone induced p65-Ser536 phosphorylation (**Figure 6A**), reflective of increased NF-κB activation by HMPV. However, pretreatment of MDMs with HMPV prior to LPS-stimulation did not impair p65-Ser536 phosphorylation relative to LPS-stimulation alone (**Figure 6A**).

In a complementary test of NF-κB activation we measured protein levels of IκBα and IκBβ, proteins that bind to and inhibit NF-κB. Stimulus-induced degradation of IκBα and IκBβ is required for activation of NF-κB (33). Immunoblotting revealed that LPS stimulation lead to reduction of both IκBα and IκBβ levels, further suggesting that NF-κB was activated by LPS. In HMPV-preinfected cells, LPS-mediated degradation of IκBα and IκBβ was not impaired relative to LPS treatment alone (**Figure 6A**). Collectively, these results show that HMPV preinfection does not suppress LPS-mediated signaling leading to NF-κB activation, suggesting that reduced NF-κB activation is not responsible for reduced transcription of IL-1β by LPS.

We found that HMPV preinfection profoundly enhanced IFN-β induction by LPS (**Figure 2C**). Corroborating this observation, we found that phosphorylation of TBK1-Ser172 and IRF3-Ser396 was markedly enhanced in LPS-stimulated MDMs that had been preinfected with HMPV (**Figure 6B**). Collectively, these results are consistent with the effect of HMPV on LPS-stimulated expression of NF-κB and IRF3-dependent genes (TNF-α, IL-6 and IFN-β; **Figure 2**).

In addition to NF-κB, the hypoxia inducible factor-1α (HIF-1α) regulates transcription of IL-1β (35). To address if HIF-1α activation was impaired by HMPV preinfection we analyzed nuclear levels of HIF-1α. We found that while LPS lead to slightly increased nuclear levels of HIF-1α, HIF-1α nuclear levels were not reduced in MDMs that had been preinfected with HMPV or IFN-β treatment (**Figure 6C**; one sample was cropped from the image and the uncropped image is included as **Figure S5**). This suggests that impaired HIF-1α function does not appear to be responsible for the reduced IL-1β transcription found in HMPV-LPS treated macrophages.

### HMPV-LPS treatment upregulates the repressive mark H3K27me3 at the *IL1B* promoter

Histone lysine methylation has been implicated in modulating cytokine output in macrophages (36, 37). H3K27me3 (trimethylated histone 3 lysine 27) is a repressive mark that is associated with inactive genes (38). Since we observed that IL-1β expression was suppressed in HMPV-LPS treated cells, we hypothesized that HMPV infection may lead to increase H3K27me3 methylation at the *IL1B* promoter, thereby repressing IL-1β transcription. To test this, macrophages were infected with HMPV and subsequently stimulated with LPS, followed by chromatin immunoprecipitation (ChIP) of H3K27me3 and qRT-PCR analysis. A specific sequence of the *IL1B* promoter centered around 250 bp upstream of the transcription start site (*IL1B*-250) was targeted, as this sequence contains a NF-κB binding site critical for IL-1β transcription (39, 40).

**Figure 7:**
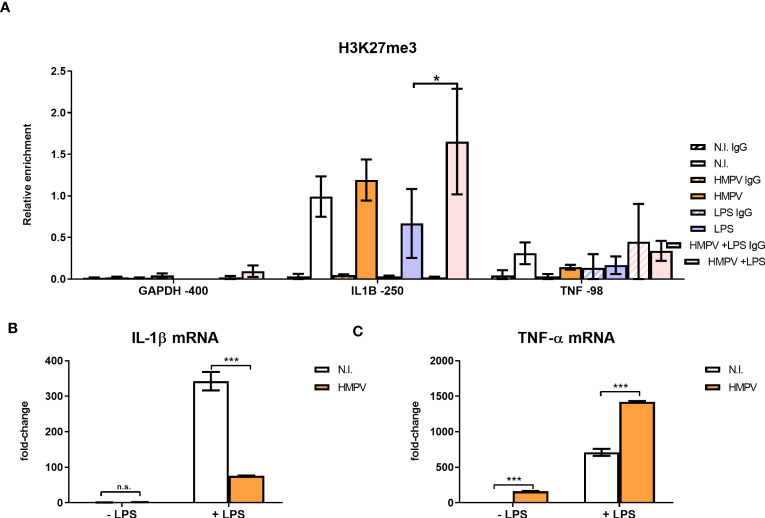
HMPV-LPS treatment upregulates the repressive mark H3K27me3 at the IL1B promoter. **(A–C)** Human macrophages were incubated with HMPV or medium and then stimulated with LPS for 2 h. Cells were either harvested for ChIP or qRT-PCR analysis **(B, C)**. **(A)** Isolated chromatin was subjected to ChIP for H3K27me3. The material recovered was analyzed by qRT-PCR using primer-probe sets specific for the indicated sequences. Numbers indicate sequence position relative to transcription start site. Target sequence enrichment for each single experiment was calculated as relative enrichment (percentage of input normalized against LPS GAPDH -400bp). Data is presented as mean of three independent biological replicates. Error bars represent SEM (3 experiments). **(B, C)** Expression of IL-1β **(B)** and TNF-α mRNA **(C)** was determined by qRT-PCR. Error bars represent SD of three technical replicates. Statistical analysis: two-way ANOVA with *post hoc* Tukey’s multiple comparisons test: ∗p < 0.05; ∗∗∗p < 0.001; n.s., not significant.

Interestingly, H3K27me3 levels at *IL1B*-250 were increased in macrophages stimulated with HMPV-LPS compared to cells treated with LPS alone (**Figure 7A**). This suggests that H3K27me3 accumulation at the *IL1B* promoter has a role in suppressing IL-1β transcription after HMPV infection. In contrast, LPS alone slightly decreased H3K27me3 at the *IL1B*-250 compared to untreated cells (**Figure 7A**). Overall, the observed the magnitude of the H3K27me3 mark at the *IL1B* promoter for all tested stimuli matched the respective IL-1β mRNA expression levels found in these samples (**Figure 7B**).

Because we found that TNF-α expression (in contrast to IL-1β) was increased in HMPV-LPS treated cells, we next examined H3K27me3 mark levels at the *TNF* promoter. To determine enrichment of TNF sequences after ChIP of H3K27me3 we quantified a sequence centered around 98 bp upstream of the transcription start (TNF -98). This sequence contains the CRE/κ3-NFAT site important for binding of the so-called “TNF enhanceosome” (41). Compared to the *IL1B* promoter, H3K27me3 levels at the CRE/κ3-NFAT site of the *TNF* promoter were low (**Figure 7A**; right part), with a tendency towards reduced H3K27me3 at TNF -98. This corroborates our finding of increased (i.e. not repressed) TNF-α mRNA expression in HMPV-LPS-treated macrophages (**Figure 7C**). Our results suggest that HMPV specifically promotes H3K27me3 at the NF-κB binding site of the *IL1B* promoter and not at the *TNF* promoter. We therefore suggest that this H3K27me3 may contribute to the reduced IL-1β induction that we observe in HMPV-LPS treated macrophages. Although these hypotheses should be tested in more detail in future experiments, the data suggests that introduction of H3K27me3 at the NF-κB target site of the IL-1β promoter in MDMs could provide a molecular explanation of our observations of a differential effect of HMPV on LPS-triggered IL-1β versus TNF-α.

## Discussion

**Figure 8:**
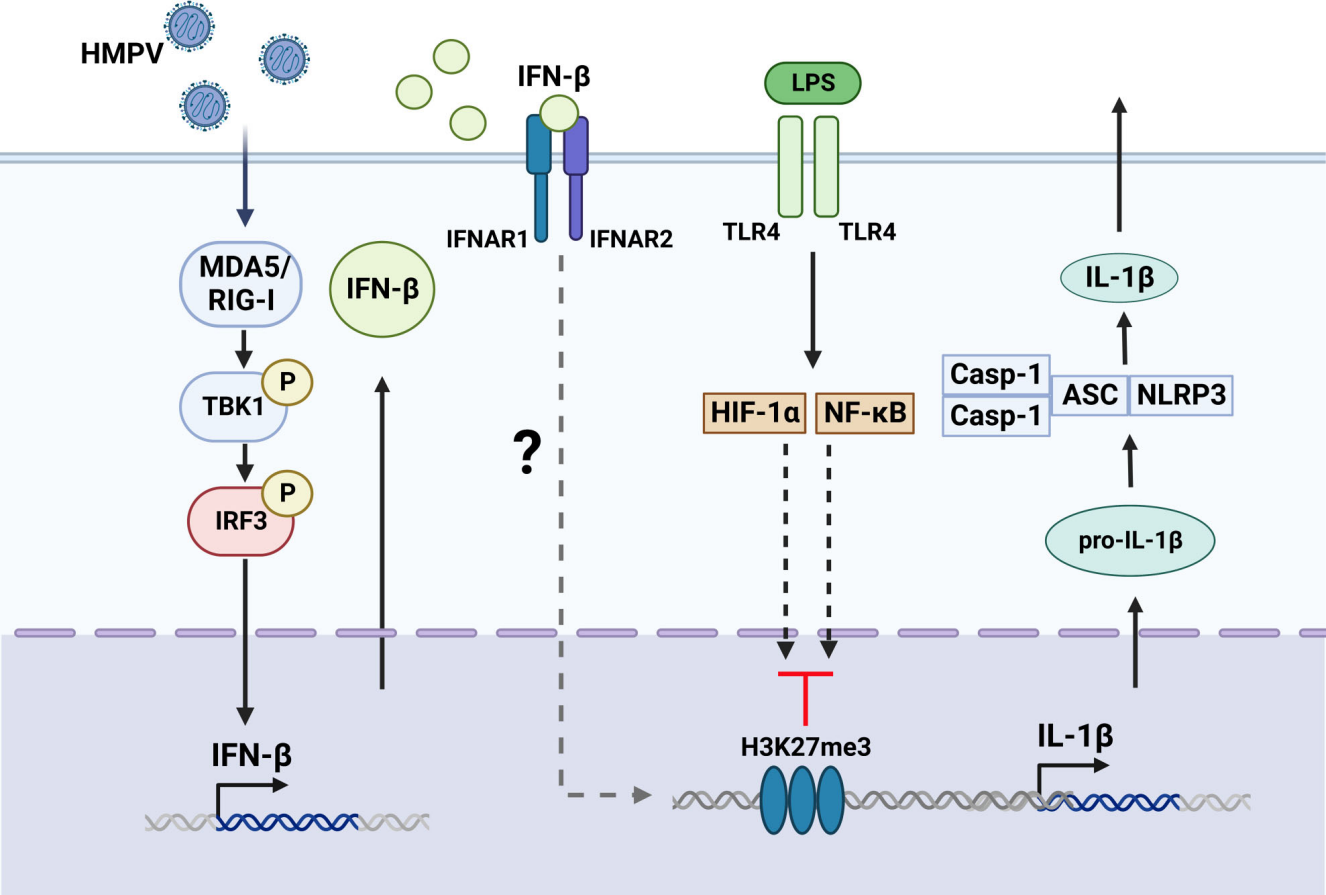
Proposed model for HMPV-mediated repression of LPS-stimulated IL-1b transcription. Based on our results we propose that HMPV stimulates TBK1-dependent IFN-β induction that acts via IFNAR-dependent mechanisms to repress IL-1β transcription upon subsequent exposure to LPS. Activation of NF-κB pathway and HIF-1α was not impaired by HMPV preinfection. We speculate the HMPV via IFN-β may introduce the repressive mark H3K27me3 at the *IL1B* promoter, thereby reducing LPS-triggered IL-1β at the transcriptional regulatory level. The Figure is created with BioRender.com.

Our study revealed that the critical human airway pathogen HMPV represses IL-1β transcription in response to LPS or heat-killed *Pseudomonas aeruginosa* and *Streptococcus pneumonia* via a mechanism mediated by TBK1-IFN-β-IFNAR (schematically illustrated in **Figure 8**). Thus our results provide new insight on the effect of HMPV on the inflammatory state of human macrophages and the molecular mechanisms by which HMPV can impact the outcome of subsequent bacterial infections.

Although HMPV has been reported to facilitate secondary bacterial, e.g. pneumococcal, infections in mice and to play a role in co-infections in the human population (8, 42, 43), the molecular mechanisms that underlie this susceptibility are poorly understood. We found that HMPV impaired bacterially induced IL-1β production in an IFN-β-dependent manner. This is similar to what was observed for influenza virus and *S. pneumoniae*-induced IL-1β (44). Since IL-1β is critical in orchestrating an adequate innate immune response against bacteria, determining molecular mechanisms regulating the impact of IFN-β on IL-1β is of high relevance for understanding and targeting virus-bacterial co-infections. IFN-β has been linked to control of IL-1β production in an earlier study by Guarda et al. (45) in which cells treated with recombinant IFN-β had limited IL-1β secretion due to reduced enzymatic activation of the NLRP3 inflammasome, thereby impairing its maturation. In contrast, our study identifies that HMPV-induced IFN-β conditions human cells to lower transcription of IL-1β. Collectively, the earlier and our studies suggest that IFN-β may repress IL-1β production by distinct mechanisms that might depend on the biological context, e.g. if produced during the setting of a viral infection or not. In this regard, we speculate that the existence of multiple mechanisms targeting IL-1β production may reflect the importance of IL-1β in antibacterial defense mechanisms and the complex regulation of IL-1β expression. Importantly, our results establish a novel and additional mechanism by which viruses and IFN-β may impair IL-1β expression and impact the host immune state. Similarly to our study, Lai et al. (43) found significant reduction of IL-1α (and other neutrophil chemoattractants) in HMPV-bacterial co-infection in mice. Moreover, a recent study in humans administered with live attenuated influenza vaccine and *S. pneumonia* reported that expression of IL-1β was reduced, whereas expression of the IRF3/IFN-β-driven cytokine IP-10 was increased (12). This is consistent with our findings of enhanced IRF-3 pathway activation in HMPV-LPS-treated macrophages. Most importantly these combined results suggest a critical role for the monocyte-macrophage lineage in human innate immune control of pneumococcus after virus infection, reflecting the importance of studying human macrophages in relation to virus-bacterial co-infections.

LPS-mediated signaling to IRF3 and NF-κB has been extensively studied and have been found to be impaired upon repeated LPS exposure through tolerance mechanisms (46). In contrast, we found these tolerance mechanisms are not triggered and that LPS-triggered activation of the NF-κB, HIF-1α and IRF3 pathways were not impaired in HMPV-infected macrophages. This suggests that other mechanisms than attenuated signaling to NF-κB and HIF-1α may be responsible for reduced IL-1β.

We found that HMPV impairs IL-1β transcription and leads to deposition of repressive H3K27me3 marks at the *IL1B* promoter. The repressive histone modification H3K27me3 has not previously been linked to virus-mediated impairment of IL-1β expression or antibacterial defense mechanisms. Methylation of H3K27 has been implicated in establishing and maintaining latency of the DNA viruses, such as human cytomegalovirus and herpes simplex virus, via H3K27me3 modifications of viral chromatin (47, 48). However, it has still not been determined if and how viruses utilize H3K27me3 to modulate host chromatin and gene responses. We observed that the repressive histone mark H3K27me3 at the *IL1B* promoter was increased in HMPV-LPS-treated cells compared to LPS-stimulated cells and this pattern of H3K27me3 levels correlated with IL-1β mRNA expression. Of note, others have shown that LPS-stimulation of macrophages can lead to acquisition of H3K27ac marks, known to be associated with actively transcribed genes at the *IL1B* promoter (49). Interestingly, both modifications compete for the same lysine residue (50). Thus, the HMPV-induced increase of H3K27me3 might prevent LPS-induced activation of IL-1β transcription by blocking acquisition of H3K27ac marks and these epigenetic marks may represent competing processes, an important question that will be addressed in future studies. Interestingly, the repressive mark H3K27me3 has earlier been found to be present at the *IL1B* promoter in cell lines that do not express IL-1β (40). In contrast, H3K27me3 was lacking in THP-1 monocyte cells that are able to induce high amounts of IL-1β.

Our results show that IFN-β mediates the suppression of IL-1β transcription by HMPV. IFN- β-mediated repression occurred within the same timeframe as reduced IL-1β transcription. It has been previously reported that influenza and coronavirus may exert epigenetic regulation of antibacterial genes in an IFN-dependent manner (51, 52). In these studies, the lysine methyltransferase SETDB2 repressed a range of NF-κB target genes such as IL-1β, TNF-α and IL-6 by histone trimethylation of H3K9. In contrast, we found a selective effect of HMPV on LPS-stimulated IL-1β mRNA relative to proinflammatory TNF-α and IL-6. Taken together, this suggests that a complementary mechanism distinct from SETDB2-mediated inflammatory repression (involving H3K27me3) occurs during HMPV infection, supported by our observation of increased H3K27me3 at the *IL1B* promoter.

We suggest that upon HMPV infection of human macrophages a mechanism of repressive mark gain additional to the previously identified SETDB2-dependent mechanism is initiated that results in increased H3K27me3 at the IL-1β promoter. As EZH2 and SUZ12 in Polycomb Repressive Complex 2 (PRC2) play a key role in H3K27me3 it is possible that the observed H3K27me3 increase at the IL-1β promoter is PRC2 complex mediated. We speculate that IFN-β via IFNAR signaling can increase H3K27me3 levels perhaps via PRC2 action specifically at the *IL1B* promoter, representing an interesting topic to establish in future studies. Regarding the longevity of the suppressive effect of HMPV on LPS-IL1β we found that suppression was initiated between 6 and 18 hours after infection and this correlated with increased viral replication and IFN-β induction. One could speculate that the effect of HMPV on IL-1β transcription disappears if HMPV levels or IFN-β is reduced. However, H3K27me3 is also regulated by H3K27me3 demethylases. Hence, the longevity of the repressive effect of HMPV on IL-1β expression is likely to depend on restrained IFN-β signaling and regulation of components controlling H3K27me3 deposition and removal.

With regards to an IFN-dependent epigenetic mechanism, it was recently reported that IFN-β via JAK-STAT-mediated IFN-stimulated gene factor 3 (ISGF3) controls deposition of active histone marks at a subset of IFN-stimulated genes (53). In parallel with this we propose that IFN-stimulated factors may regulate deposition of repressive histone marks like H3K27me3 at specific genes, e.g. IL-1β during viral infections. As with IFN-stimulated genes for which viruses have distinct profiles of ISGs they induce, viruses may show virus-specfic effects on IFN-mediated epigenetics control. Understanding how different viruses and IFNs control histone marks during viral infections is highly relevant for understanding susceptibility to secondary infections.

In conclusion, our findings identify a novel potential mechanism for the increased susceptibility to bacterial infections after HMPV infections and point to the possible involvement of the repressive mark H3K27me3 in HMPV/IFN-β-mediated modulation of antibacterial gene expression.

## Data availability statement

The raw data supporting the conclusions of this article will be made available by the authors, without undue reservation.

## Author contributions

All authors contributed to the study concept and design of different parts of the study. SL, NM, AW and TS conducted the experiments, collected and analysed the data. All authors contributed to the article and approved the submitted version.
