# Supplementary figures and images for: Human metapneumovirus driven IFN-β production antagonizes macrophage transcriptional induction of IL1-β in response to bacterial pathogens
Source: Front Immunol. 2023 Jun 26;14:1173605. doi: 10.3389/fimmu.2023.1173605 (PMC10330783; doi:10.3389/fimmu.2023.1173605)

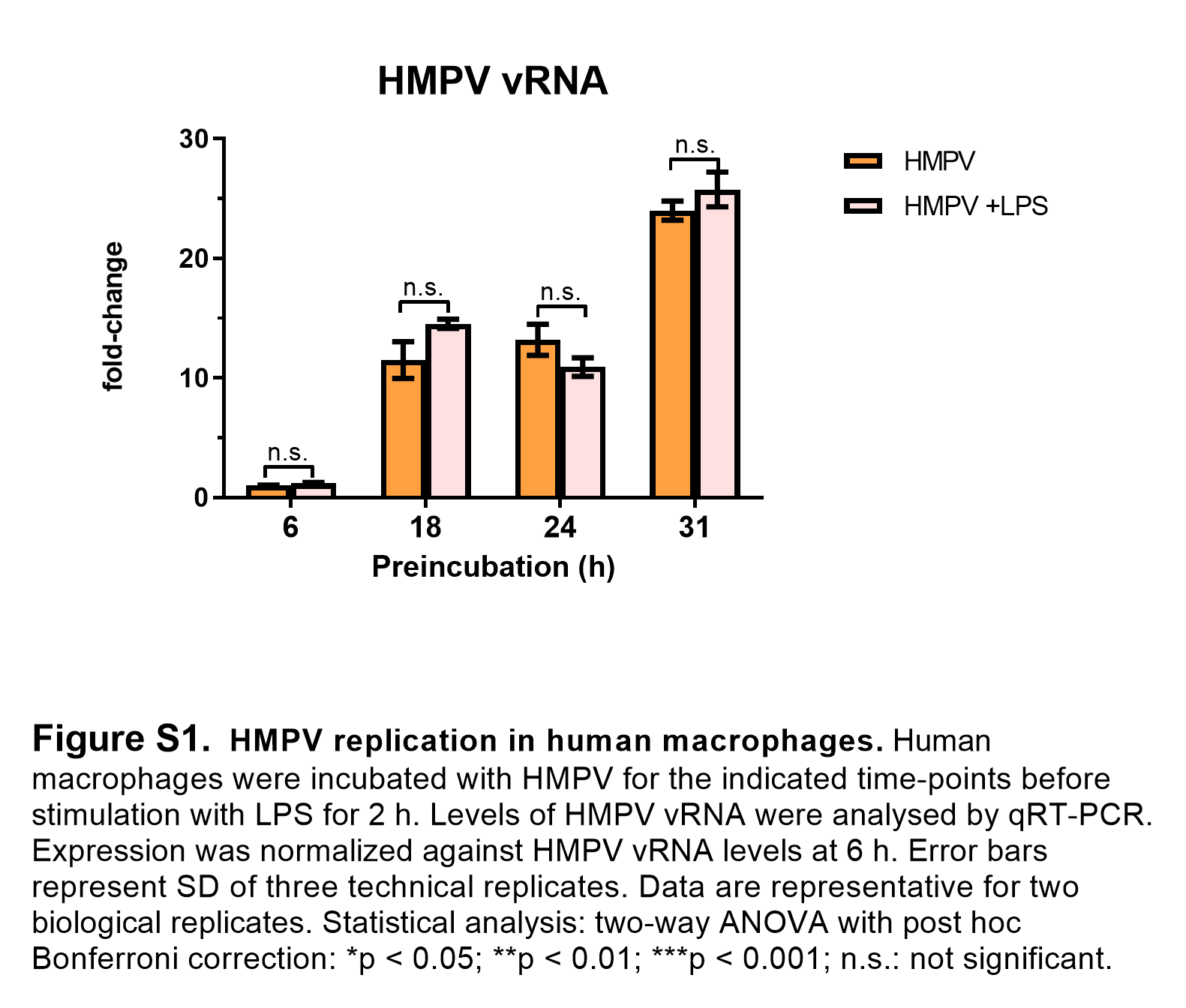

Supplement: Supplementary file 1 [file Image_1.tif]

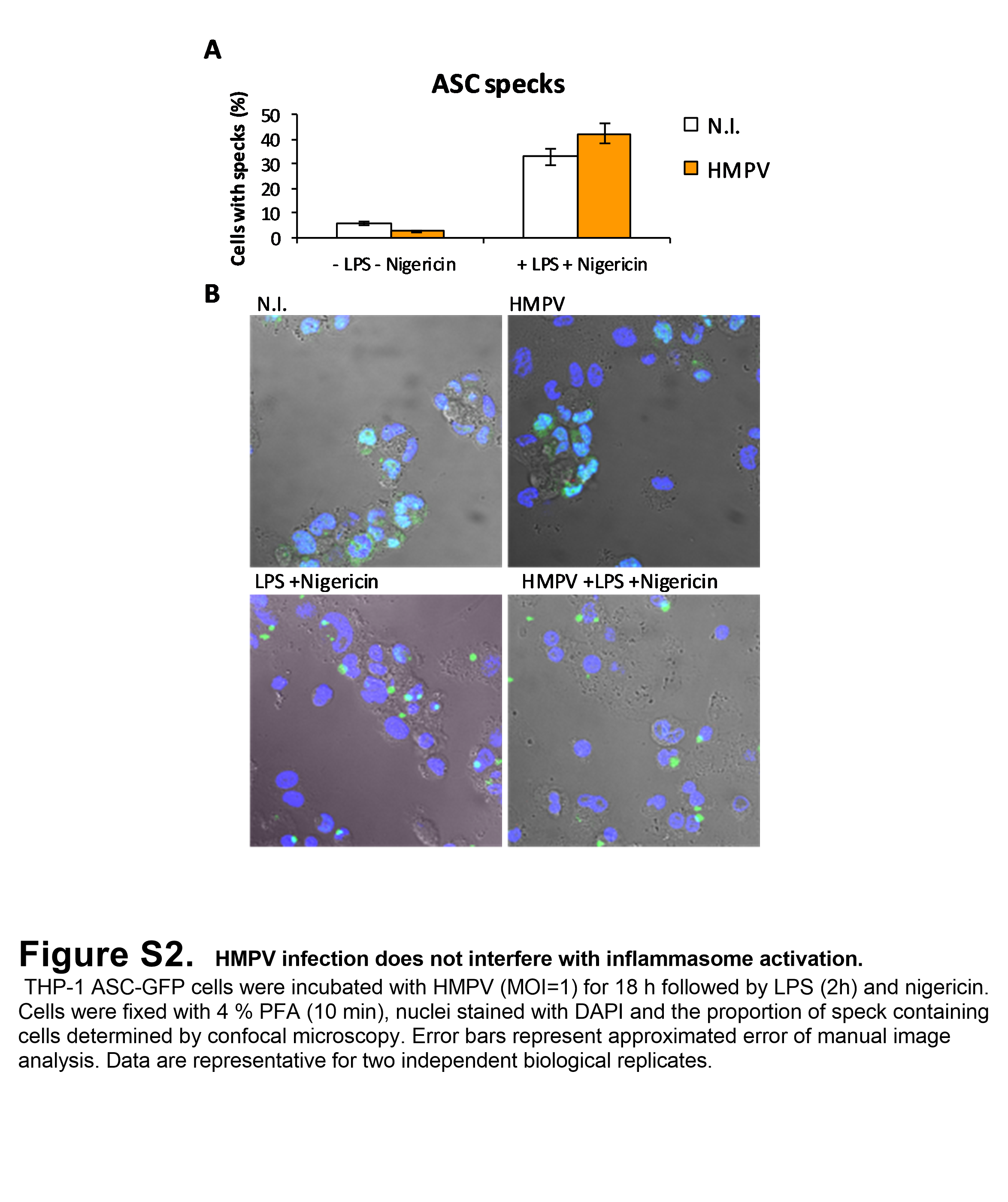

Supplement: Supplementary file 2 [file Image_2.tif]

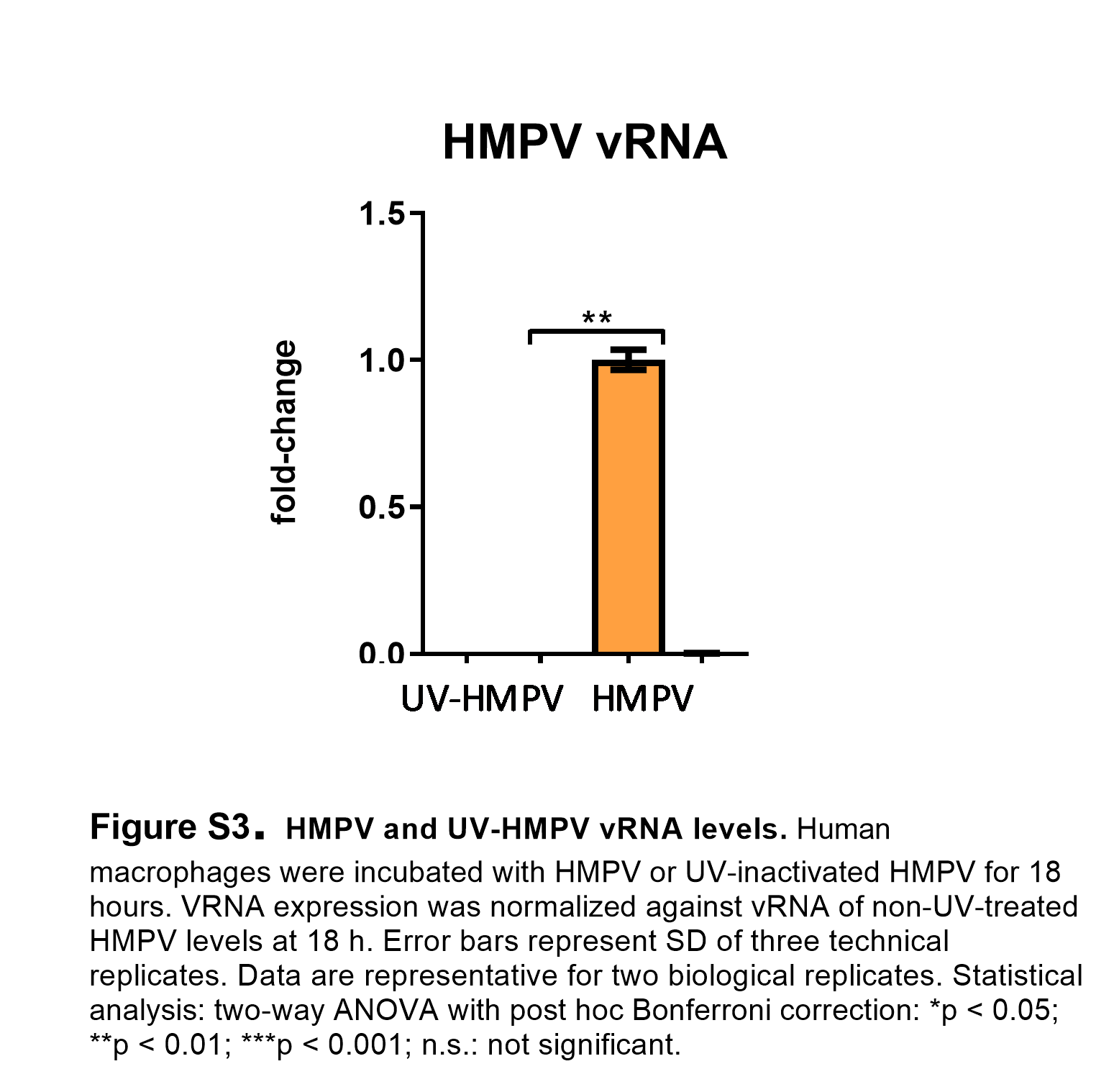

Supplement: Supplementary file 3 [file Image_3.tif]

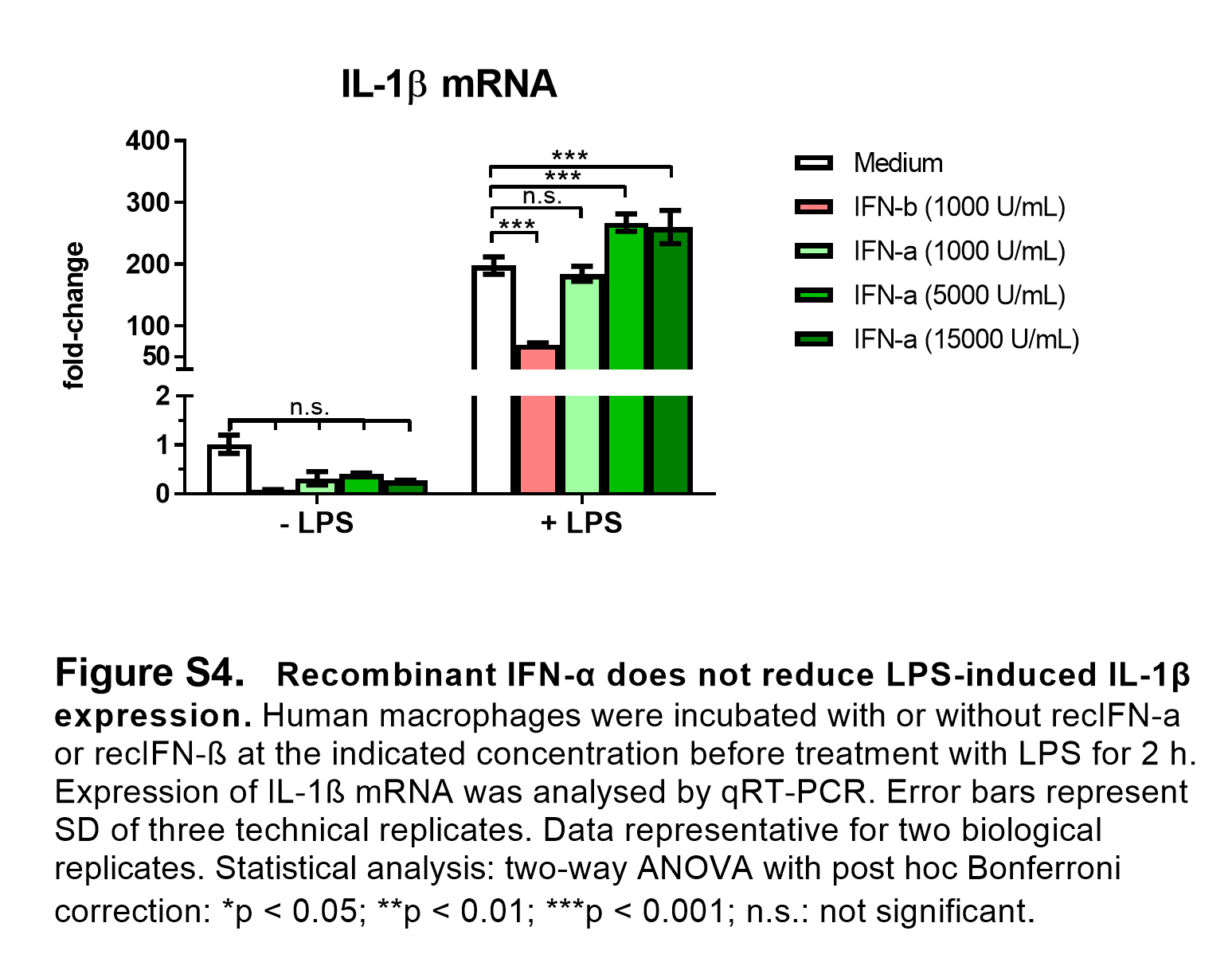

Supplement: Supplementary file 4 [file Image_4.tif]

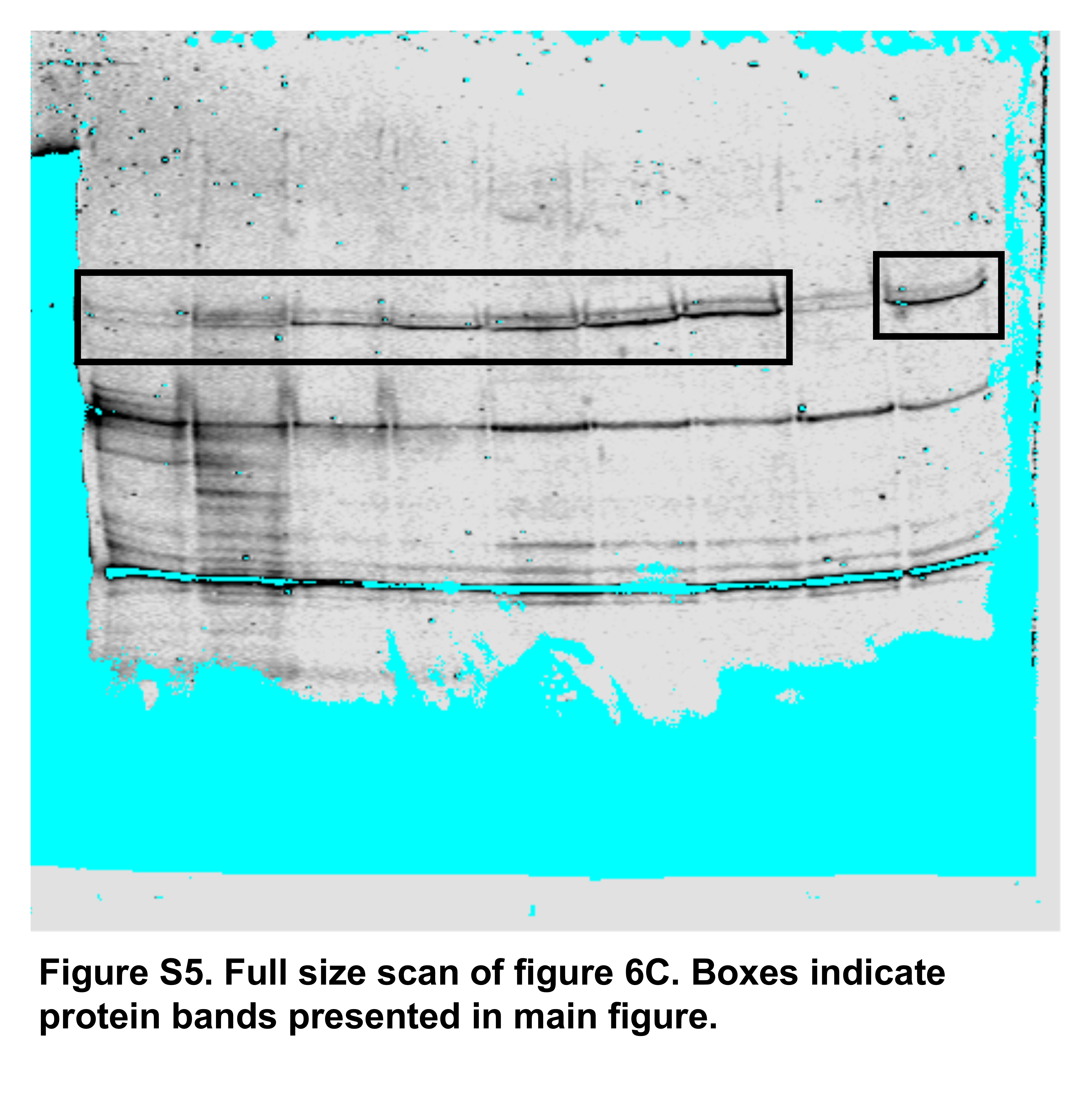

Supplement: Supplementary file 5 [file Image_5.tif]
